# Copper-Containing Anti-Biofilm Nanofiber Scaffolds as a Wound Dressing Material

**DOI:** 10.1371/journal.pone.0152755

**Published:** 2016-03-30

**Authors:** Jayesh J. Ahire, Melanie Hattingh, Deon P. Neveling, Leon M. T. Dicks

**Affiliations:** Department of Microbiology, University of Stellenbosch, 7602 Matieland (Stellenbosch), South Africa; Laurentian, CANADA

## Abstract

Copper particles were incorporated into nanofibers during the electrospinning of poly-D,L-lactide (PDLLA) and poly(ethylene oxide) (PEO). The ability of the nanofibers to prevent *Pseudomonas aeruginosa* PA01 and *Staphylococcus aureus* (strain Xen 30) to form biofilms was tested. Nanofibers containing copper particles (Cu-F) were thinner (326 ± 149 nm in diameter), compared to nanofibers without copper (CF; 445 ± 93 nm in diameter). The crystalline structure of the copper particles in Cu-F was confirmed by X-ray diffraction (XRD). Copper crystals were encapsulated, but also attached to the surface of Cu-F, as shown scanning transmission electron microscopy (STEM) and transmission electron microscopy (TEM), respectively. The copper particles had no effect on the thermal degradation and thermal behaviour of Cu-F, as shown by thermogravimetric analysis (TGA) and differential scanning calorimeter (DSC). After 48 h in the presence of Cu-F, biofilm formation by *P*. *aeruginosa* PA01 and *S*. *aureus* Xen 30 was reduced by 41% and 50%, respectively. Reduction in biofilm formation was ascribed to copper released from the nanofibers. Copper-containing nanofibers may be incorporated into wound dressings.

## Introduction

Copper is an essential metal and is required in small quantities in many metabolic processes [1, 2]. Under controlled conditions, copper plays an important role in wound healing by enhancing the expression of integrin, and stabilizing fibrinogen and collagen formation [1, 3–7]. Excessive use of copper is toxic, as it generates free radicals by the Fenton and Haber-Weiss reaction, which may lead to lipid peroxidation and cell death [8, 9]. However, copper-transporting adenosine triphosphatases (Cu-ATPases), such as ATP7A and ATP7B in humans, maintain the homeostasis and excrete copper through the intestine, liver and mammary glands [10–12]. In a more recent study with bacterial cells, it was shown that copper nanoparticles causes protein oxidation and DNA degradation [13].

In ancient times, copper was used to sterilize water and treat burn, skin and ear infections [14]. Intra-uterine copper devices have been in use for many years [1] and in 2008 the US Environmental Protection Agency (USEPA) permitted the use of copper alloys to control microbial growth [15]. Most pathogens, including strains of *Pseudomonas aeruginosa*, *Enterobacter aerogenes*, *Staphylococcus aureus*, methicillin-resistant *S*. *aureus* (MRSA), vancomycin-resistant *Enterococcus* (VRE), *Clostridium difficile*, *Salmonella enterica*, *Campylobacter jejuni* and *Escherichia coli* 0157:H7, are killed when exposed to the surfaces of copper and copper alloys [16–22]. Copper oxide-impregnated dressings enhanced wound healing in genetically engineered diabetic mice [1].

Exopolysaccharides (EPS) safeguard cells from antibiotics, antimicrobial peptides and harsh environmental conditions [23, 24]. At least two reports published last year suggested treatment of biofilms with copper-containing nanoparticles [25, 26]. To the best of our knowledge, the incorporation of copper into nanofibers and its effect on biofilms has not been reported.

Silver is well known for its antimicrobial activity and silver nanoparticles (Ag-NPs) incorporated into nanofiber dressings have been used in wound dressings [9, 27–29]. In this paper we describe the electrospinning of copper particles into biodegradable nanofibers prepared from a 1:1 combination of poly-D,L-lactide (PDLLA) and poly(ethylene oxide) (PEO). Antimicrobial properties of copper-containing nanofibers (Cu-F) were tested against biofilms of *P*. *aeruginosa* PA01 and a methicillin-resistant *S*. *aureus*, strain Xen 30.

## Materials and Methods

### Materials

Copper powder was supplied by TraceXtec (TraceXtec, Pty., Ltd., South Africa). PDLLA (Mw 75−120 kDa) and PEO (Mw 200 kDa), *N*,*N*-dimethylformamide (DMF), 3-(4,5-dimethylthiazol-2-yl)-2,5-diphenyltetrazolium bromide (MTT), Dulbecco’s modified Eagle's medium (DMEM), Hams-F12, fetal bovine serum (FBS), hydrocortisone, insulin, cholera toxin and PenStrep were obtained from Sigma-Aldrich (St. Louis, MO, USA). The LIVE/DEAD^®^ Baclight^TM^ Bacterial Viability kit and FilmTracer^TM^ SYPRO^®^ Ruby Biofilm Matrix Stain were from Thermo Fisher Scientific (Massachusetts, USA). All reagents were of analytical grade.

### Preparation of copper-containing nanofibers

A 24% (w/v) 1:1 combination of PDLLA and PEO, dissolved in DMF, was heated at 40°C to produce a homogenous solution. Copper powder was suspended in the polymer solution to the final concentration of 150 mg ml^−1^. Initial studies have shown that this was the maximum concentation of copper particles that could be electrospun into nanofibers. Nanofibers were electrospun as described by Heunis et al. [30]. PDLLA/PEO nanofibers without copper powder served as control.

### Characterization of nanofibers

Nanofibers were sputter-coated with carbon and their surface morphology visualised by scanning electron microscopy (SEM), using a FEI Nova nanoSEM 230 (FEI, Hillsboro, Oregon, USA). A total of 100 image points were measured to determine the average diameter of the nanofibers. ImageJ Software (version 1.46, Scion Corporation, Maryland, USA) was used. Transmission electron microscopy (TEM) images were recorded on a Philips Tecnai TF20 (FEI).Copper particles in nanofibers was visualised using a FEI Nova nanoSEM 230 (FEI), equipped with an X/Max Oxford energy-dispersive X-ray (EDX) detector (Oxford Instruments, Oxfordshire, UK). INCA software (Berkshire, UK) was used to analyse the EDX spectra. Scanning transmission electron microscopy (STEM) images were recorded using the FEI Nova nanoSEM 230, equipped with STEM detector. Physical phases were observed by X-ray diffraction (XRD) using a Bruker AXS D8 Advance X-ray diffractometer (Bruker AXS, Frankfurt, Germany), equipped with a Vantec-1 position sensitive detector (Cu-Kα radiation at λ = 1.5406 Å) and operated in locked coupled mode. The X-ray tube was operated at 40 mA and 40 kV. Readings were recorded at a scanning rate of 88 sec/step, with a step size of 0.05° in a 2θ range that extended from 15° to 95°. The thermal stability, melting endotherms and crystallization exotherms of nanofibers were measured by thermogravimetric analyser (TGA) and differential scanning calorimeter (DSC), as described by Ahire et al. [31].

### *In vitro* antimicrobial activity

Active-growing cells of *P*. *aeruginosa* PA01 and *S*. *aureus* Xen 30 (10^5^ CFU ml^–1^) were each spread-plated onto Muller-Hinton agar (Fluka, Sigma-Aldrich Pty, Ltd., Aston Manor, South Africa). Sections of 0.5 cm^2^ copper-containing nanofibers (Cu-F) and nanofibers without copper (CF) were placed on the surface of the spread plates and the plates incubated at 37°C for 24 h. The diameter of growth inhibition zones was measured in millimetres.

### Biofilm formation in the presence of nanofibers

*Pseudomonas aeruginosa* PA01 and *S*. *aureus* Xen 30 (Caliper Life Sciences, Hopkinton, USA) were stimulated to form biofilms, as described by Ahire and Dicks [32, 33]. Sections of Cu-F (0.5 cm^2^) were placed in 24 wells of a 96-well plastic round-bottom multidish (Corning, Sigma-Aldrich Pty, Ltd.). Another 24 wells received CF (0.5 cm^2^). Cell suspensions of *P*. *aeruginosa* PA01 and *S*. *aureus* Xen 30 were prepared in sterile tryptone soy broth (TSB, Biolab Diagnostics, Biolab, Midrand, South Africa) to 7.3 ± 0.07 log _10_ CFU ml^–1^ and 6.6 ± 0.11 log _10_ CFU ml^–1^, respectively. Two-hundred microliters of the *P*. *aeruginosa* PA01 cell suspension was transferred to 12 wells containing Cu-F and 12 wells containing CF. Twelve wells with Cu-F and 12 wells with CF were each inoculated with 200 μl *S*. *aureus* Xen 30 cell suspension. Twelve wells without nanofibers were inoculated with 200 μl *P*. *aeruginosa* PA01 and another 12 wells with the same volume *S*. *aureus* Xen 30. All plates were statically incubated at 37°C for 48 h.

At specific time intervals, nanofibers and planktonic cells were carefully removed from the wells and discarded. The wells were then washed twice with sterile distilled water and air dried. Total biofilm formation was determined by staining the wells with crystal violet [32, 33]. Optical density readings were taken at 595 nm.

### Number of viable cells in biofilms

Sterile PBS, pH 7.3 (100 μl) was added to wells immediately after washing with sterile distilled water. The cells were carefully suspended by using a sterile glass rod, the suspension serially diluted in PBS and plated onto TSA (Biolab). Colonies were counted after 24 h of incubation at 37°C.

### Confocal laser scanning microscopy (CLSM) of biofilms

Immediately after washing the cells with sterile distilled water, biofilms were carefully removed from the wells with a sterile pipette tip and transferred to 100 μl sterile PBS (pH 7.3). The biofilms were stained with the LIVE/DEAD^®^ Baclight^TM^ Bacterial Viability kit (Thermo Fisher Scientific, Massachusetts, USA) and FilmTracer^TM^ SYPRO^®^ Ruby Biofilm Matrix Stain (Thermo Fisher Scientific), as recommended by the suppliers. A Carl Zeiss LSM780 confocal microscope (Carl Zeiss, Göttingen, Germany) was used to study the stained biofilms. Images were processed by using the ZEN 2011 imaging software (Carl Zeiss).

### Surface characterization of biofilms

Wells that contained biofilms were washed and dried as described elsewhere. After drying, the wells were cut out with a sharp blade and images of the wells taken with a FEI Nova nanoSEM 230 (FEI). The topology of the surfaces was studied using a Nanosurf Easyscan 2 atomic force microscope (AFM; Nanosurf AG, Switzerland). The AFM was operated in static force mode, equipped with a CantAI-G cantilever. The P-gain was adjusted to 2800, rotation to 45° and tip voltage to zero. Images were recorded in the range of 10 μm (*x* and *y*) and AFM parameters were evaluated using the SPIP 3D image processing 6.3.6 software (Image Metrology A/S, Hørsholm, Denmark).

### Controlled release of copper

Sections of Cu-F (10 mg) were each submersed in 1 ml sterile deionized water and incubated at 25°C. The nanofiber mat was transferred to sterile deionized water, every 2 h, for up to 24 h. Release of copper from the nanofiber mats was studied with inductively-coupled plasma mass spectrometry (ICP-MS), using an Agilent 7700 ICP-MS (Agilent Technologies, München, Germany).

### *In vitro* cytotoxicity

The cytotoxicity of nanofibers was performed as described by Ahire et al. [34]. Briefly, breast epithelial cells (MCF-12A) were cultured in DMEM and Hams-F12 (1:1), supplemented with 10% FBS, 0.5 μg ml^–1^ hydrocortisone, 10 μg ml^–1^ insulin, 100 ng ml^–1^ cholera toxin and 1% (v/v) PenStrep. Cells were seeded in 12 × 24-well NEST tissue culture plates (Nest Biotechnology, Wuxi Jiangsu, China) to 8.0 × 10^4^ per well and incubated at 37°C in humidified atmosphere (5% CO_2_). After 24 h, the medium in wells was replaced with UV-sterilized Cu-F (0.5 cm^2^), CF (0.5 cm^2^), and fresh culture media (negative control, NC), respectively. The plates were incubated for a further 24 h, after which cell viability was determined using the MTT assay.

### Statistical analysis

All experiments were performed in triplicate. Values are expressed as mean ± standard deviation (SD). One-way analysis of variance (ANOVA) was calculated by using GraphPad Prism (6.07-Trial, GraphPad Software Inc,CA, USA). A *P* value < 0.05 or less was considered statistically significant.

## Results and Discussion

### Characterization of nanofibers

**Fig 1:**
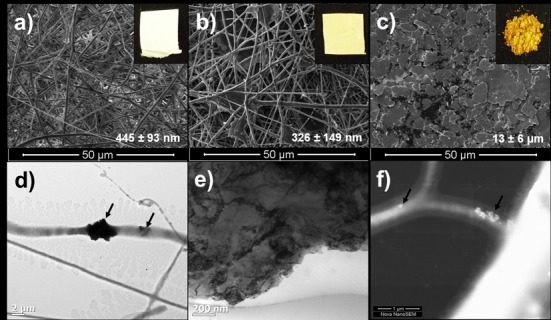
Scanning electron microscopy (SEM) images of (a) control nanofibers (CF), (b) copper-containing nanofibers (Cu-F) and (c) copper particles before electrospinning. Digital images of the electrospun nanofiber mats and copper powder are shown as inserts. The TEM image of Cu-F (d) shows the encapsulation of copper crystals (right arrow) in nanofibers and the attachment of crystals on the surface (left arrow) of the fibers. Image (e) is the same as (c), but visualised with TEM. Image (f) is the same as (d), but recorded with STEM and shows the inclusion of copper crystals of different sizes in Cu-F (two arrows).

Copper particles in the PDLLA/PEO solution accelerated a liquid jet towards a grounded collector, due to an increase in conductance. Similar findings were reported with the presence of copper nanoparticles during the spinning of polyurethane nanofibers [35]. This explains the smaller diameter of Cu-F (average 326 ± 149 nm) compared to CF (average 445 ± 93 nm). The choice of solvent, distance between needle and collector has a major effect on fiber morphology. As the solvent evaporates, the liquid jet is stretched to many times its original length to produce continuous, ultrathin, fibers of the polymer [36]. Apart from a more gold-coloured Cu-F, the structure of Cu-F and CF were identical when visualised under the SEM (Fig 1A and 1B). Images recorded with TEM clearly showed copper particles encapsulated in nanofibers (Fig 1D, right arrow), but also attached to the surface of the fibers (Fig 1D, left arrow). Images obtained with STEM confirmed the inclusion of copper particles in the fibers (Fig 1F, both arrows). No selected area electron diffraction pattern (SAED) was detected for copper. The irregular shape of the copper particles, recorded with SEM and TEM, before electrospinning, is shown in Fig 1C and 1E, respectively. The irregular shape and size of the copper particles resulted in an uneven distribution of the particles in the polymer and thus also the nanofibers. The irregular morphology of the copper particles is ascribed to the method used to produce particles from metal sheets.

**Fig 2:**
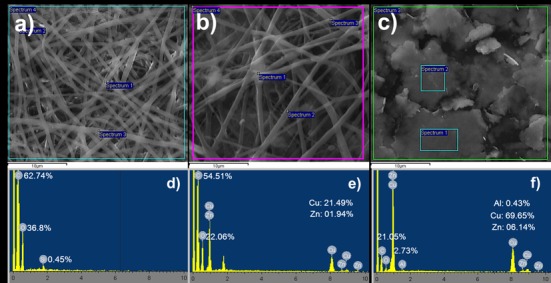
SEM-energy-dispersive X-ray (EDX) analysis of (a) nanofibers without copper (CF), (b) copper-containing nanofibers (Cu-F) and (c) copper particles. The EDX scan spectra of selected areas pointed out in images (a), (b) and (c) are shown in (d), (e) and (f), respectively. The percentages shown for each of the elements are the mean of three selected areas from SEM images.

Analyses with SEM-energy-dispersive X-ray (EDX) of Cu-F (Fig 2B) and copper particles (Fig 2C) revealed the presence of C, O, Cu, and Zn (Fig 2E and 2F). In addition, trace quantities of Al (0.43 ± 0.41 wt. %) were also detected in copper particles (Fig 2C and 2F). Analyses of CF showed C and O as major elements, with small quantities (0.45 ± 0.12 wt. %) of Si (Fig 2A and 2D).

**Fig 3:**
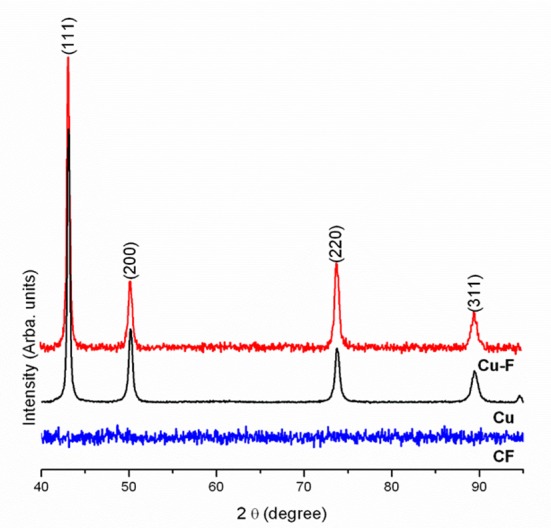
X-ray diffraction (XRD) patterns of nanofibers with copper particles (Cu-F), nanofibers without copper particles (CF) and copper particles (Cu).

Diffraction peaks at 2*θ* values of 43.12°, 50.10°, 74.0°, and 89.6° recorded with XRD corresponded to (111), (200), (220) and (311) crystal planes (Fig 3) of the JCPDS database [37]. These results confirmed the crystalline structure of copper in the nanofibers.

**Fig 4:**
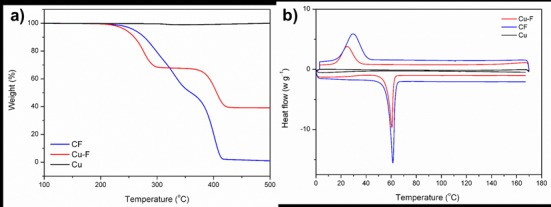
(a) Thermal behaviour of control nanofibers (CF), copper-containing nanofibers (Cu-F) and copper particles (Cu). (b) Differential scanning calorimetric (DSC) thermograms of CF, Cu-F and Cu.

With TGA analyses (Fig 4A), two distinct weight losses were observed for CF and may be attributed to the PDLLA (50 wt. %) and PEO (50 wt. %) homo-polymers, respectively [31]. The addition of copper particles had no major effect on the thermal degradation of the nanofibers. Similar to the CF, two weight losses were visible until 40% of the original weight remained. This implies that the sample had a copper content of 40 wt. %, as neat copper was stable throughout the entire temperature range. No thermal events were observed in DSC for neat copper within the operating temperature range. The melting endotherms and crystallization exotherms recorded for Cu-F were similar to those observed for CF, indicating that the copper particles had no effect on the thermal behaviour of the samples (Fig 4B).

### *In vitro* antimicrobial activity

Copper-containing nanofibers (Cu-F) placed on the surface of plates inhibited the growth of *P*. *aeruginosa* PA01 (inhibition zone diameter: 5 ± 1.1 mm) and methicillin resistant *S*. *aureus* Xen 30 (inhibition zone diameter: 12 ± 1.5 mm). Nanofibers without copper particles (CF) did not inhibit the growth of the two species and confirmed previous findings [31–34, 38, 27]. The smaller inhibition zone recorded for *P*. *aeruginosa* PA01 is ascribed to the strain’s natural resistance to copper [39]. Resistance to copper may be due to less negatively charged peptidoglycan in the cell wall [40]. The outer membrane of Gram-negative bacteria act as barrier to protect the cell from the penetration of biocides and antibiotics [41]. Accumulation of high levels of copper in the cytoplasm induces a Fenton and Haber-Weiss reaction that mediates lipid peroxidation and cell death [8, 42]. The oxidation product of metallic copper, i.e. Cu^2+^, is known to oxidise proteins and degrade DNA [13]. Similar mechanisms of antimicrobial activity have been reported for Ag-NPs [43]. Silver particles electrospun into PDLLA: PEO inhibited *P*. *aeruginosa* PA01 and methicillin resistant *S*. *aureus* Xen 30 [27]. The difference in antimicrobial activity observed between copper and silver particles incorporated into nanofibers may be due to strain sensitivity or the rate at which the particles are released from the fibers [44].

### Biofilm formation in the presence of nanofibers

**Fig 5:**
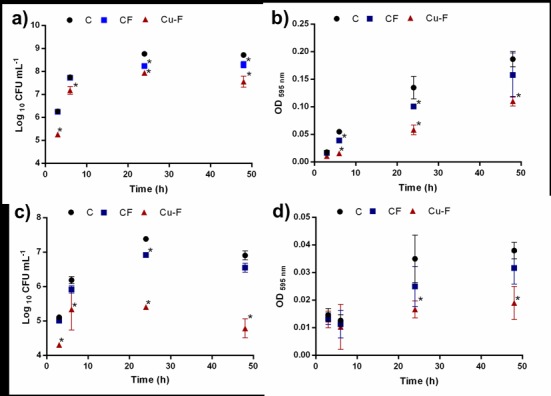
Changes in viable cell numbers and biofilm formation by *P*. *aeruginosa* PA01 and *S*. *aureus* Xen 30 in the presence of copper particles electrospun into nanofibers (Cu-F) and nanofibers without copper particles (CF). Cells not treated with copper and not cultured in the presence of nanofibers serves as control (labelled c). Incubation was at 37°C. Readings were taken at 3, 6, 24 and 48h. (a) Number of viable cells of *P*. *aeruginosa* PA01, (b) total biofilm formation by cells of *P*. *aeruginosa* PA01, (c) number of viable cells of *S*. *aureus* Xen 30 and (d) total biofilm formation by cells of *S*. *aureus* Xen 30. Biofilm formation was determined by staining with crystal violet and recording OD readings at 595 nm. Data points presented are the average of three independent experiments (mean ± standard deviation). * *p< 0*.*05*.

**Fig 6:**
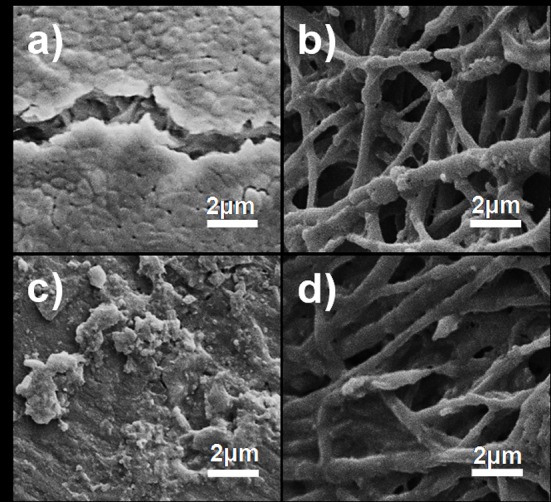
Scanning electron microscopy (SEM) images of nanofibers collected from 48-h-old biofilms. Images (a) and (c) show cell growth of *P*. *aeruginosa* PA01 and *S*. *aureus* Xen 30, respectively, on the surface of CF. Images (b) and (d) show cell growth of *P*. *aeruginosa* PA01 and *S*. *aureus* Xen 30, respectively, on the surface of Cu-F.

Viable cell numbers recorded for *P*. *aeruginosa* PA01 and *S*. *aureus* Xen 30 were after 48 h approximately 5% less in the presence of CF, compared to cells grown in the absence of nanofibers (Fig 5A and 5C, respectively). The decline in cell numbers is attributed to adhesion of the two strains to nanofibers (CF), as shown in Fig 6A and 6C. Growth of *P*. *aeruginosa* PA01 declined with approximately 13% in the presence of Cu-F (Fig 5A) and growth of *S*. *aureus* Xen 30 with approximately 31% (Fig 5C). After 48 h in the presence of Cu-F, biofilm formation of *P*. *aeruginosa* PA01 decreased with approximately 41% (Fig 5B) and that of *S*. *aureus* Xen 30 with approximately 50% (Fig 5D), compared to biofilm formation in the absence of nanofibers and copper. Very few cells of *P*. *aeruginosa* PA01 and *S*. *aureus* Xen 30 adhered to Cu-F (Fig 6B and 6D). Based on these findings, copper that diffused from Cu-F inhibited cell growth and biofilm formation of *S*. *aureus* Xen 30 and *P*. *aeruginosa* PA01.

In a recent study, growth inhibition and biofilm formation of MRSA and *P*. *aeruginosa* were shown when the cells were treated with copper-containing nanoparticles (Cu-NPs) [25, 26]. Christina et al. [26] reported a 88% decrease in *P*. *aeruginosa* biofilm biomass and a 90% decrease in *S*. *aureus* biofilm biomass when treated with Cu-NPs. We did not determine changes in biomass, but recorded a drastic decline in biofilm formation based on crystal violet staining (41% for *P*. *aeruginosa* PA01 and 50% *S*. *aureus* Xen 30).

### Confocal imaging of biofilms

**Fig 7:**
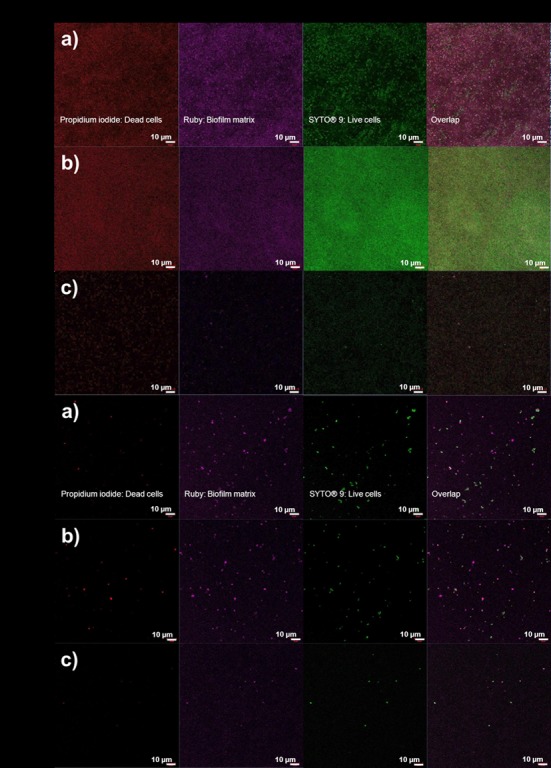
LIVE/DEAD® Baclight^TM^ and FilmTracer^TM^ SYPRO® Ruby Biofilm Matrix Stain of 48-h-old biofilms of *P*. *aeruginosa* PA01 (A) and *S*. *aureus* Xen 30 (B). For each strain, images were taken of biofilms that formed in (a) the absence of nanofibers and copper, (b) nanofibers without copper particles (CF) and (c) copper-containing nanofibers (Cu-F). The images in column 1 is after propidium iodide staining, column 2 after ruby staining and column 3 after SYTO® 9 staining. The image in column 4 is an overlap of all stains.

Staining with LIVE/DEAD^®^ Baclight^TM^ and FilmTracer^TM^ SYPRO^®^ Ruby Biofilm Matrix Stain showed only few viable cells in the presence of Cu-F (Fig 7Ac and 7Bc), compared to many more viable cells detected in the presence of CF (Fig 7Ab and 7Bb). The intense fluorescence recorded with ruby stain of *P*. *aeruginosa* PA01 cells, incubated in the absence of nanofibers and copper, and in the presence of CF, may be due to the presence of a high concentration of glycoproteins, phosphoproteins, lipoproteins, calcium-binding proteins and fibrillar proteins in the biofilm. These proteins are difficult to stain. The intensity of fluorescence was directly proportion to the number of cells present in the biofilms.

### Characterization of biofilms by SEM and AFM

**Fig 8:**
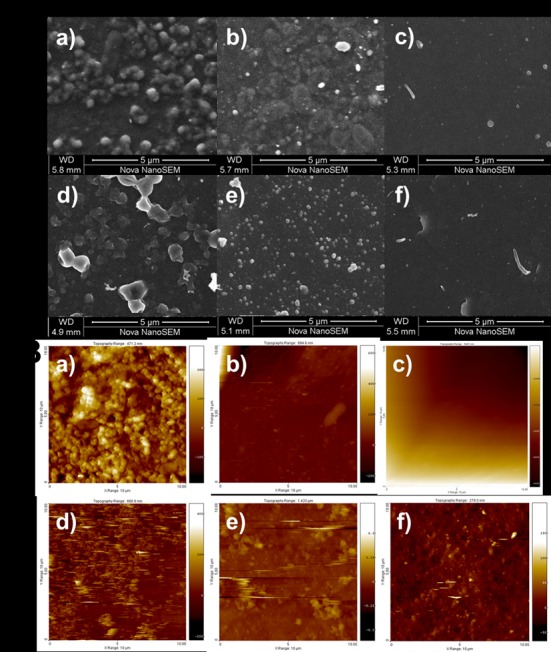
A: Scanning electron microscopy (SEM) images of *P*. *aeruginosa* PA01 biofilms (a, b and c) and *S*. *aureus* Xen 30 biofilms (d, e and f) that formed after 48 h on the polymer surfaces of the wells. B: Atomic force microscopy (AFM) images of *P*. *aeruginosa* PA01 biofilms (a, b and c) and *S*. *aureus* Xen 30 biofilms (d, e and f) that formed after 48 h on the polymer surfaces of the wells. Images a and d were taken from biofilms that formed in the absence of nanofibers and copper particles, b and e from biofilms that formed in the presence of CF, and c and f from biofilms that formed in the presence of Cu-F.

Images of 48-h-old biofilms of *P*. *aeruginosa* PA01 and *S*. *aureus* Xen 30 recorded with SEM and AFM clearly showed inhibition of biofilm formation in presence of Cu-F (Fig 8Ac and f, and 8Bc and f). Surface colonization of cells with hydrocolloids and EPS was clearly visible in untreated (Fig 8Aa and d, and 8Ba and d) and CF-treated (Fig 8Ab and e, and 8Bb and e) biofilms. The development of biofilm architecture is highly important for the exchange of nutrients, pathogenicity and resistance to antimicrobial compounds [45]. Our results confirmed that Cu-F controlled biofilm formation by inhibiting bacteria and the formation of extracellular matrix.

### Controlled release of copper

**Fig 9:**
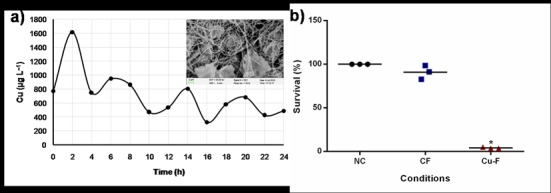
A: Release of copper from PDLLA/PEO nanofibers. A SEM image of the electrospun nanofibers after the release of copper particles is shown in the insert. B: Percentage of MCF-12A breast epithelial cells that survived exposure to CF and Cu-F. Sterile culture medium served as negative control (NC). Data points presented are the average of three independent experiments (mean ± standard deviation). * *p* < 0.05.

An initial 773 μg of copper release was recorded, followed by a burst release of 1616 μg of copper after 2 h (Fig 9A). After 4 h, fluctuations in copper release were observed, with a stepwise decline after every 6 h (Fig 9A). This is ascribed to the gradual disintegration of the hydrophilic PEO from PDLLA in the PDLLA: PEO matrix and the formation of an amorphous matrix, as shown by Heunis and co-workers [38]. Exposed copper particles in degraded nanofibers, shown in SEM images (insert, Fig 9A), supports the findings. The rate at which copper is released from solid surfaces depends on the releasing media. Low release rates were reported in water and phosphate buffer, whereas much higher release rates were recorded in Tris-Cl buffer and spent M17 medium [44]. The sudden release of high concentrations of copper during the first 2 h is important to control infections and restrict biofilm formation by killing more planktonic cells. The burst release of copper is similar to that reported for nisin, 2,3-dihydroxybenzoic acid, Ag-NPs, and ciprofloxacin incorporated into PDLLA/PEO nanofibers [27, 31–34, 38].

### Cytotoxicity of Cu-F

Survival of MCF-12A breast epithelium cells was significantly affected in the presence of Cu-F (Fig 9B). No changes in the viability of MCF-12A were recorded when the cells were exposed to CF, confirming the safety of PDLLA/PEO nanofibers [34]. The 3% survival of cells in the presence of Cu-F indicated a higher sensitivity of breast epithelial cells towards copper, or a maximum release of copper particles in the media used in this study. Exposure of copper to skin induced far less adverse reactions [46]. Further *in vitro* tests on keratinocytes and *in vivo* tests with rats or mice need to be performed.

## Conclusion

Despite irregularity observed in the shape of the copper particles, they were successfully incorporated into PDLLA and PEO nanofibers and remained in crystal form. The encapsulated copper particles had no effect on the thermal behaviour of the nanofibers, even at an inclusion of 40 wt. %. Biofilm formation by *P*. *aeruginosa* PA01 and *S*. *aureus* Xen 30 may be controlled by the release of copper from the nanofibers. Furthermore, very few cells of *P*. *aeruginosa* PA01 and *S*. *aureus* Xen 30 adhered to the copper-containing nanofibers, suggesting that these nanofibers may be used to control initial cell growth in biofilms and possibly also infected wounds. Only a few breast epithelial cells (3%) survived exposure to copper-containing fibers, which suggests that the levels of copper released from the nanofibers were highly toxic to cells in tissue culture. Other studies have shown that copper concentrations at these levels have no adverse reaction when applied on human skin [46].
